# The detection of pathological parathyroid glands is facilitated by identifying vascular features on ultrasound: the potential benefit of a low-frequency vascular probe

**DOI:** 10.1007/s12020-024-03986-y

**Published:** 2024-08-08

**Authors:** Sanne Høxbroe Michaelsen, Mette Bay, Oke Gerke, Ole Graumann, Anders Rørbæk Madsen, Christian Godballe, Steen Joop Bonnema, Viveque Egsgaard Nielsen

**Affiliations:** 1 Research Unit for ORL – Head & Neck Surgery and Audiology, Odense University Hospital, Odense, Denmark; 2 Department of Clinical Research, University of Southern Denmark, Odense, Denmark; 3 OPEN, Open Patient data Explorative Network, Odense University Hospital, Odense, Denmark; 4 Department of Nuclear Medicine, Odense University Hospital, Odense, Denmark; 5 Department of Clinical Medicine, Aarhus University, Aarhus, Denmark; 6 Department of Nuclear Medicine, Aarhus University Hospital, Aarhus, Denmark; 7 Department of Endocrinology, Odense University Hospital, Odense, Denmark

**Keywords:** Primary hyperparathyroidism, Parathyroid, Ultrasound, Pre-operative imaging

## Abstract

**Purpose:**

To evaluate the potential benefit of adding a low frequency vascular probe to the conventional pre-operative ultrasound examination of patients with primary hyperparathyroidism.

**Methods:**

A prospective cohort of 136 patients with primary hyperparathyroidism underwent a conventional ultrasound examination of the neck with a high frequency ( > 10 MHz) linear ultrasound probe, followed by an add-on examination with a low frequency vascular probe. For each ultrasound probe, and for every potential parathyroid lesion, the presence of a feeding vessel, a polar placement of the feeding vessel, and the presence of a vascular arch was recorded.

**Results:**

A total of 146 ultrasound lesions were evaluated for vascularity by each probe. For both ultrasound probes, the odds of a hyperfunctioning parathyroid gland being correctly identified increased with the number of visible vascular features. The vascular probe identified a significantly higher number of vascular features among ultrasound true positive glands compared with the conventional probe (*p* < 0.0001). Among histopathologically verified pathological parathyroid glands, the vascular probe identified 20% more feeding vessels, 27% more polar placements of the feeding vessel, and 65% more vascular arches than the high frequency probe. However, the diagnostic confidence score for true positive glands did not differ significantly between the probes (*p* = 0.11).

**Conclusion:**

The addition of a low frequency vascular probe increases the number of visible vascular features in hyperfunctioning parathyroid glands, which facilitates their preoperative detection. Whether or not this can increase the diagnostic confidence of ultrasound examiners has yet to be substantiated.

## Introduction

Most patients with primary hyperparathyroidism (PHPT) have a single hyperfunctioning parathyroid gland (89%), while a minority have multiple gland hyperplasia (6%), or double adenomas (4%) [1]. The definitive cure for PHPT is surgical removal of all hyperfunctioning parathyroid tissue [2]. Surgical planning is facilitated by pre-operative imaging, which attempts to determine both the location and number of enlarged parathyroid glands. If the pre-operative localization is successful, surgeons can perform a minimally invasive procedure in patients with single gland disease or plan the least extensive bilateral procedure for patients with multiple gland disease [3].

Pre-operative imaging regimens vary between institutions, but ultrasound (US) is the recommended technology, as it enables a simultaneous evaluation of the thyroid gland [2, 4]. Although US has a high sensitivity for the detection of enlarged parathyroid glands in experienced hands [5], many diagnostic set-ups involve a parallel acquisition of US and a radiation-based imaging modality, such as ^99m^Tc-sestamibi subtraction scintigraphy, single-photon emission computed tomography (SPECT), SPECT/CT, four-dimensional CT, or positron emission tomography (PET)-CT with a radiolabelled tracer [2, 6, 7]. To avoid superfluous imaging procedures, however, a number of centers with experienced parathyroid ultrasound examiners have abandoned parallel imaging acquisition in favor of a stepwise regimen, in which an upfront US examination determines the need for additional procedures [8, 9].

Upfront US places high demands on the quality of the US examination and the ability of the US probe to visualize the typical sonographic features of a parathyroid adenoma: A hypoechoic and sharply delineated lesion with an enlarged feeding artery entering the pole and continuing around the periphery in a 90 to 270 degree vascular arch [10, 11]. In general, high frequency ( > 10 MHz) US probes are used to evaluate hyperfunctioning parathyroid glands and current guidelines on parathyroid ultrasound do not suggest using a separate transducer for the detection of vascular features [10, 12]. However, when our tertiary referral center recently performed a study comparing US and contrast-enhanced ultrasound (CEUS) with scintigraphy [13], we experienced that the lower frequency range (3–11 MHz) peripheral vascular probe dedicated to CEUS could reveal parathyroid vascular features that had not been visible with the high frequency probe, even without the addition of a contrast agent. Therefore, we hypothesized that the conventional ultrasound examination of patients with PHPT would benefit from the addition of a lower frequency dedicated vascular probe in terms of revealing more vascular features.

## Materials and methods

This prospective, paired cohort study is based on a larger non-inferiority study of 172 patients, which compares conventional US and CEUS with scintigraphy, as previously reported [13]. The study is registered at ClinicalTrials.gov (NCT04305561) and has been approved by the Regional Committees on Health Research Ethics for Southern Denmark (project ID S-20190077). The study was carried out in accordance with the ethical principles described in the 1964 Declaration of Helsinki and its later amendments. The inclusion of the current subgroup started on December 19, 2019, and ended on February 25, 2021 at the Department of Otorhinolaryngology, Head & Neck Surgery, and Audiology at Odense University Hospital, Denmark. Participants signed an informed consent document and formed a consecutive series. The reporting guideline Strengthening the Reporting of Observational Studies in Epidemiology (STROBE) was used [14].

### Study participants

Eligible patients were adults ( ≥ 18 years) with PHPT who were referred for parathyroidectomy. Patients were excluded if they were not legally competent; were pregnant/nursing; could not read/speak Danish; had prior parathyroid or thyroid surgery; had an ongoing malignant neoplasm; did not undergo scintigraphy or CEUS in the Region of Southern Denmark; had contraindications to CEUS; or were not booked for surgery [13].

### Setup and blinding

All patients underwent a dual-tracer ^99m^technetium-pertechnetate/^99m^technetium-sestamibi subtraction scintigraphy with ^99m^technetium-sestamibi SPECT/CT (dtSM SPECT/CT) prior to an in-office consultation with one of three experienced parathyroid surgeon-sonographers. The surgeon-sonographer was blinded to the result of the dtSM SPECT/CT until all US examinations had been performed and documented.

### Notation of localization, diagnostic confidence, and vascular features

**Fig. 1:**
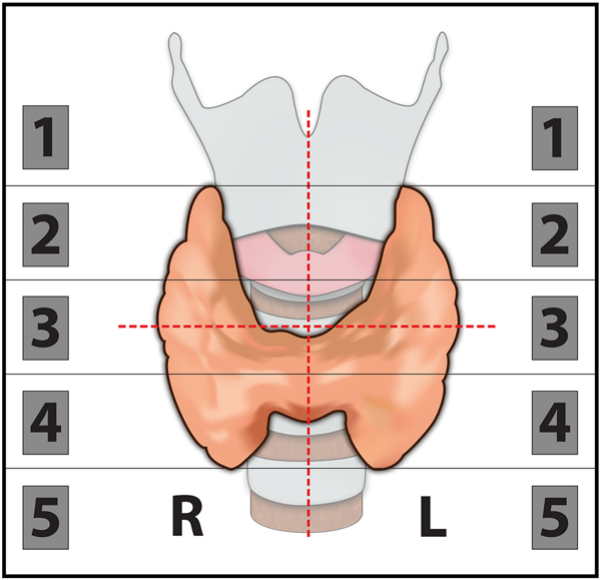
Illustration used for parathyroid localization

Each ultrasound probe had its own scoring sheet featuring a coronal illustration of the thyroid gland, which had been divided into quadrants as well as a 10-part classification (Fig. 1). Potential pathological glands were drawn on the illustration, and each predefined position was assigned a confidence score from a 5-point Likert-type scale. Low scores of 1 or 2 were assigned to imaging negative positions, and high scores of 4 or 5 were assigned to imaging positive positions. If no potential pathological gland was identified anywhere, a neutral score of 3 was assigned to all positions, categorizing the examination as non-localizing.

A high suspicion of pathology was indicated with score 5 for the given location combined with score 1 for all remaining negative positions. A moderate suspicion was indicated with score 4 for the given location combined with score 1 for all remaining negative positions. A low suspicion was indicated with score 4 for the given location combined with score 2 for all remaining negative positions.

All imaging-positive lesions were evaluated for the presence/absence of three vascular features: i. a feeding artery, ii. polar placement of the feeding artery, iii. a vascular arch.

### Conventional high frequency ultrasound

The conventional US examination was carried out on a Canon® Aplio a450 US machine, version 1.3 (Canon Medical Systems, Japan) with a PLT-1005BT (14L5) linear transducer with center frequency pre-sets of 8.0, 11.0, and 14.0 MHz. Patients were placed in a supine position with their neck extended. First, the probe was placed on a central part of the thyroid gland in the transversal plane to optimize settings. The highest frequency providing visualization of the longus colli muscle was chosen, and the depth was adjusted to show the thyroid gland and underlying longus colli muscle. The focus was set on the dorsal border of the thyroid gland, but could be adjusted once a potential pathological parathyroid gland was encountered. Scanning commenced cranial to the thyroid gland in the transverse plane and continued caudally to the clavicle, where the probe was tilted until the subclavian artery or vein was visualized. The probe was then rotated 90 degrees clockwise, and scanning continued in the longitudinal plane [15]. All potential parathyroid lesions were evaluated for vascularity with Advanced Dynamic Flow and Superb Microvascular Imaging. The location, diagnostic confidence, and number of visible vascular features were documented before the ensuing examination with the low frequency vascular probe was conducted.

### Low frequency vascular probe

The vascular ultrasound examination was performed as an add-on to conventional ultrasound and consisted of the initial steps of a CEUS examination, before the injection of contrast. Because CEUS requires a dedicated vascular probe, an integrated part of the procedure is to re-identify – with the vascular probe and before contrast injection – all suspected pathological parathyroid glands visualized during conventional ultrasound. This examination was undertaken by one of two surgeon-sonographers trained in CEUS, using a PLT-705BT (11L3) peripheral vascular linear transducer with center frequency pre-sets of 6.0, 7.0, and 8.0 MHz. Settings were optimized and suspected parathyroid lesions were re-identified and scored for diagnostic confidence and the number of visible vascular features, as described above. This additional documentation step was scored separately from the ensuing CEUS examination.

### Surgery

If a potential parathyroid adenoma had been preoperatively identified, a minimally invasive approach was chosen. If preoperative imaging results were discordant, the surgeon autonomously decided which location to target first, guided by the respective confidence scores. Two pre-incision parathyroid hormone (PTH) plasma levels were measured at the beginning of surgery. A ≥ 50% drop in plasma PTH from the highest pre-incision level, measured ten minutes after the excision of a potential parathyroid gland, signaled that no further exploration was necessary [16]. The in vivo location of excised tissue was documented according to the specifications described above (Fig. 1) combined with the Perrier classification, which is a nomenclature covering the most frequent anatomical locations of parathyroid adenomas [17]. All specimens underwent histopathological evaluation.

### Follow-up

After surgery, patients were referred to their local department of endocrinology, where they were followed with blood tests for at least six months [13]. A minority of patients (19 of 136 [14%]) finalized follow-up with their family physician after showing stable normocalcemia for a median of 122 days (IQR 85–148) at the local department of endocrinology. Individuals in this group were assumed to have remained normocalcemic if they had not been re-referred to the hospital by the end of the study, i.e. December 31, 2021.

### Outcome measures

For obvious reasons, pathological parathyroid glands that were overlooked on ultrasound could not be evaluated for vascularity. The main outcome measure was the number of vascular features identified by each probe. Exploratory analyses furthermore assessed the association between the number of visible vascular features and the number of true/false positive glands for each probe, the association between the number of visible vascular features and the diagnostic confidence, and the diagnostic confidence reached by each probe. For reference purposes, we also calculated the per-patient sensitivity ( ≥ 1 true positive quadrant per patient) and the per-quadrant sensitivity, specificity, positive predictive value, and negative predictive value. These results are available in Online Resource 1. Since a diagnosis of PHPT was an inclusion criterion, calculating the specificity per patient was not meaningful.

The reference standard consisted of the in vivo surgical localization and histopathology of the excised specimen combined with long-term cure (normocalcemia 6 months after surgery). If a specimen was located in-between two quadrants (i.e. in the middle of the thyroid gland), the 10-part classification served as a reference [13]. Patients with residual disease at follow-up were conservatively categorized as having had one pathological parathyroid gland in each quadrant at the time of surgery, representing the maximum possible number of pathological glands that the ultrasound examiner could have overlooked. A true positive quadrant had been correctly categorized on ultrasound as harboring a diseased gland (adenoma or hyperplasia on histopathology). A true negative quadrant had been correctly categorized as free from pathology in a patient who achieved long-term cure. A false positive quadrant had been categorized as harboring a diseased gland on ultrasound, but no pathological tissue was removed from it during surgery - in a patient who achieved long-term cure. A false negative quadrant had been categorized as free from pathology on ultrasound, but an adenomatous or hyperplastic gland was either excised from it during surgery or assumed in residual disease.

### Sample size

The sample size of 136 patients was predetermined by the sample size of the overall cohort [13]. Regarding the main outcome measure, we made an a priori calculation of the discordant proportions necessary to reach a power >80% and a significance level of 0.05 when using McNemar’s test, two correlated proportions, and a sample size of 136. Assuming that the conventional ultrasound probe could identify 5% of vascular features that were not visible on the vascular probe, the power would be >80%, provided that the vascular probe could identify at least 16% of vascular features not visible on the conventional ultrasound probe.

### Statistical methods

Descriptive statistics were derived according to data type; continuous variables were described with median (minimum-maximum), categorical variables with frequencies and respective percentages. The Wilcoxon signed rank test was used to compare the ultrasound probes (conventional vs. vascular) regarding the number of visualized vascular features and the diagnostic confidence of true positive glands. A logistic regression was used to assess the relationship between the number of visible vascular features and the number of true/false positive glands. A Kruskal-Wallis test and Cuzick’s test of trend were used to assess the relationship between the number of vascular features in true positive glands and the confidence score. McNemar’s test was used to compare the probes at the level of each individual vascular feature and to assess patient-based sensitivity. Quadrant-based analyses for diagnostic accuracy measures were performed as a regression of the binary outcome (e.g. for sensitivity: true positives vs. false negatives) on modality, with cluster-robust standard errors to account for the four quadrants within each patient [18]. Ninety-five percent confidence intervals were supplemented for respective point estimates. Two-sided *p*-values < 0.05 were considered statistically significant.

Study data were managed with REDCap® electronic data capture tools hosted at Odense University Hospital [19, 20]. Statistical calculations were performed in Stata/BE: release 18 (StataCorp LLC) and Microsoft Excel® 2016.

## Results

**Fig. 2:**
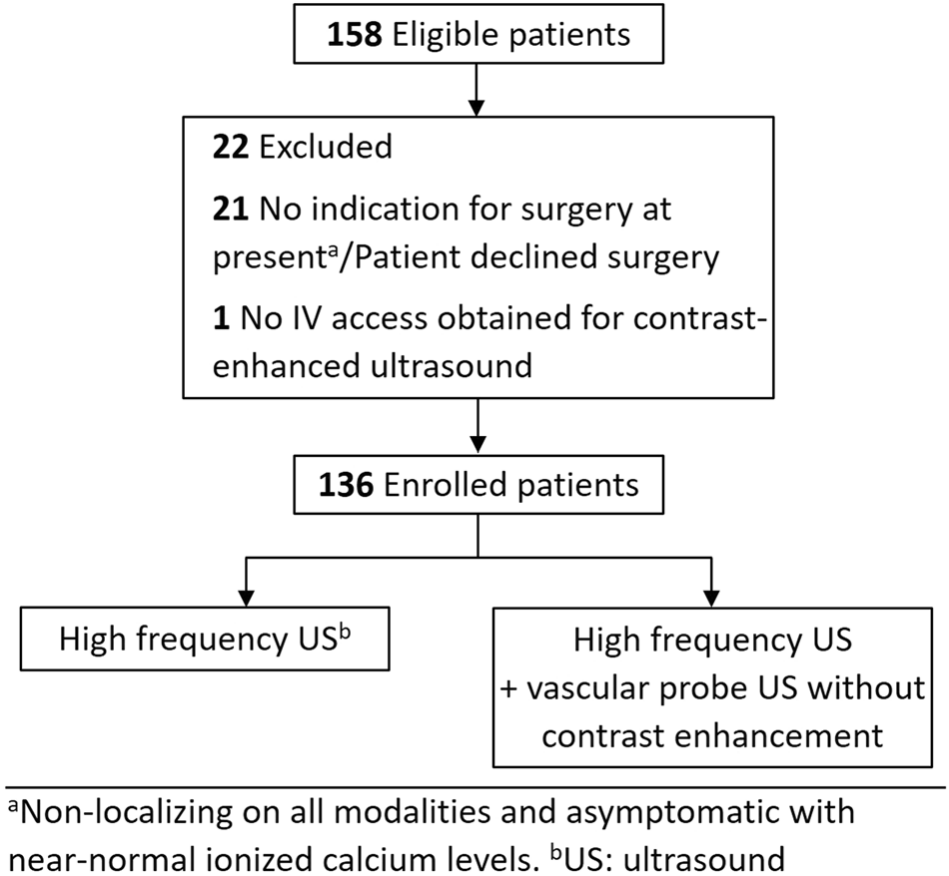
Flowchart with reasons for exclusion

Out of 158 eligible patients, 22 were excluded, leaving 136 patients for inclusion in the study (Fig. 2). Two patients (1.5%) did not achieve long-term cure, while the remaining 134 patients (98.5%) were relieved of hyperparathyroidism post-surgery. A total of 146 hypoechoic ultrasound lesions in 125 patients were considered to be “ultrasound positive” by at least one probe and consequently evaluated for vascularity. The remaining 11 patients had non-localizing ultrasound scans. Patient characteristics are listed in Table 1.

**Table 1:** Patient characteristics

	All 136 patients	125 patients with lesions evaluated for vascularity
Age, median (range), *y*	64 (27–87)	64 (27–87)
Sex, *n* (%)		
Female	113 (83.1)	107 (85.6)
Male	23 (16.9)	18 (14.4)
BMI in kg/m^2^, median (range)	26.4 (16.5–42.8)	26.2 (16.5–42.8)
No. of patients, *n* (%)		
1-gland disease	124 (91.2%)	115 (92.0%)
2-gland disease	7 (5.1%)	6 (4.8%)
4-gland disease	5 (3.7%)	4 (3.2%)
Parathyroid carcinoma	0 (0%)	0 (0%)
Thyroid pathology on ultrasound, *n* (%)		
Normal thyroid on US	77 (56.6%)	72 (57.6%)
Multinodular goiter	45 (33.1%)	39 (31.2%)
Partly intrathoracic	10 (7.4%)	8 (6.4%)
Solitary adenoma	5 (3.7%)	5 (4.0%)
Other	4 (2.9%)	4 (3.2%)
Diffuse goiter	1 (0.7%)	1 (0.8%)
Preoperative plasma ionized calcium, median (range), reference range 1.18–1.32 mmol/L at pH 7.4	1.43 (1.31–1.79)	1.43 (1.31–1.79)
Preoperative plasma parathyroid hormone^a^, median (range) [n]		
Ref. range 1.6–6.0 pmol/L	8.6 (3.0–20.0) [*n* = 84]	8.6 (3.0–20.0) [*n* = 79]
Ref. range 1.1–6.9 pmol/L	10.3 (7.0–12.3) [*n* = 6]	11.6 (7.0–12.3) [*n* = 5]
Ref. range 1.6–6.9 pmol/L	11.5 (4.9–39.0) [*n* = 28]	11.5 (4.9–39.0) [*n* = 24]
Ref. range 1.7–9.2 pmol/L	13.8 (4.6–20.1) [*n* = 17]	13.6 (4.6–20.1) [*n* = 16]
Ref. range 1.2–8.3 pmol/L	18.2 (18.2) [*n* = 1]	18.2 (18.2) [*n* = 1]
Follow-up in days, median (IQR) [range]^b^	196.5 (180–253.75) [7–429]	197 (180–278.5) [7–429]
Pathological parathyroid glands, *n*	158	146
Gland weight in mg, median (range)	445.5 (41–8305)	439 (41–8305)
Unknown weight, no. of glands^c,d^ (%)	10^c^ (6.3)	9^d^ (6.2)
High frequency ultrasound true positive glands (*n* = 116)		
Largest diameter in mm, median (range)	11 (3–35)	11 (3–35)

*BMI* body mass index (weight in kilograms/height in meters squared)

^*a*^*The referring hospitals used different parathyroid hormone assays*

^*b*^*Date of the last blood test before a patient is referred to their family physician*

^*c*^*Five presumed pathological glands in two patients with persistent hyperparathyroidism six months after surgery* + *3 glands left in situ in subtotal parathyroidectomies* + *1 intrathyroidal gland* + *1 gland with no recorded weight*

^*d*^*Five presumed pathological glands in two patients with persistent hyperparathyroidism six months after surgery* + *2 glands left in situ in subtotal parathyroidectomies* + *1 intrathyroidal gland* + *1 gland with no recorded weight*

### Vascular features

**Fig. 3:**
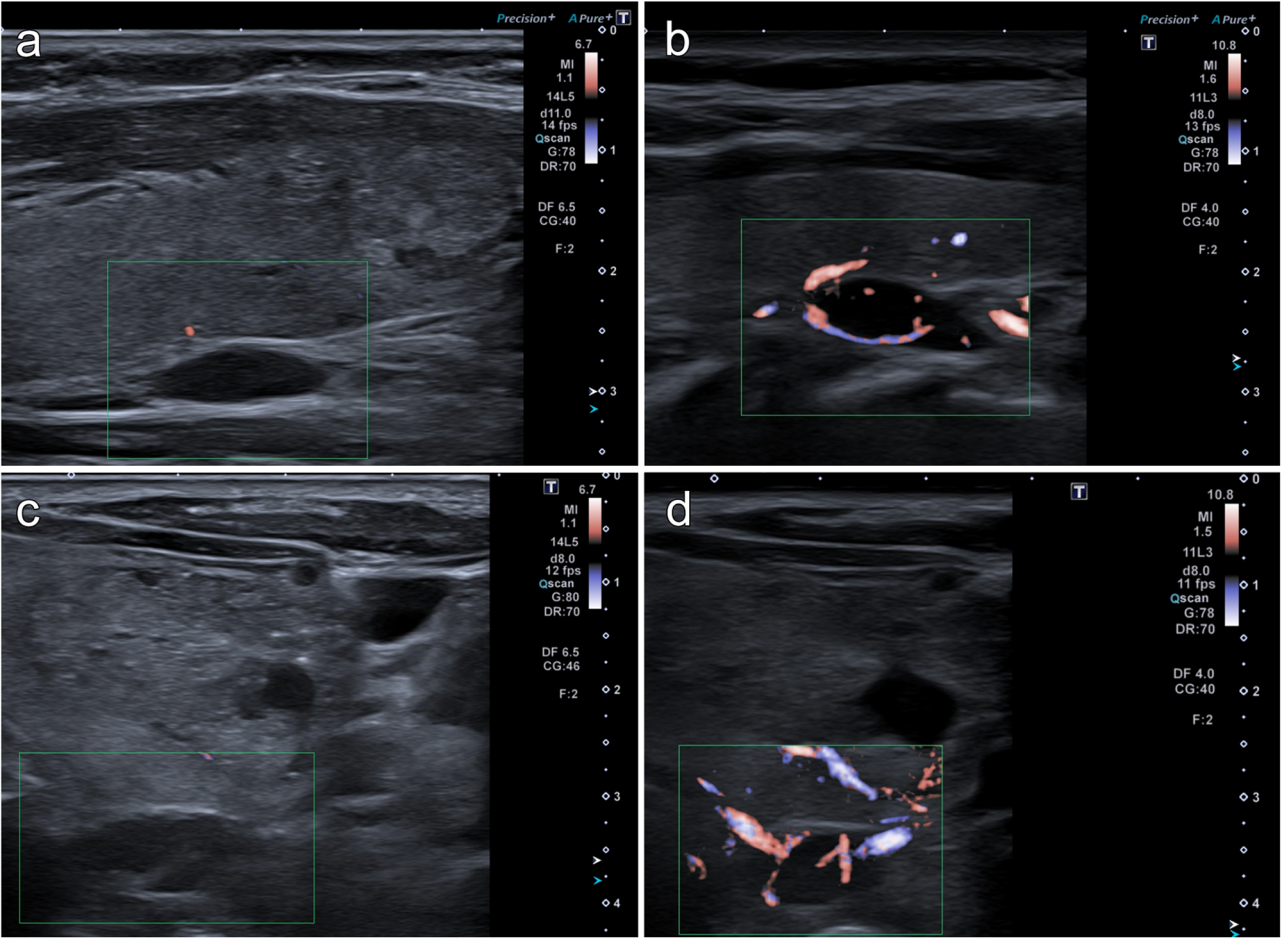
Example of two parathyroid glands **a, c** with no visible vascular features on the conventional probe. Both glands had a visible polar feeding vessel and vascular arch upon examination with the vascular probe **b, d**

For both ultrasound probes, the odds of a gland being true positive increased with the number of visible vascular features (Table 2). The vascular probe identified a significantly higher number of vascular features among ultrasound true positive glands compared with the conventional probe (*p* < 0.0001, Table 2). This was also the case when each individual vascular feature was assessed (Table 3). The vascular probe identified 20% more feeding vessels, 27% more polar placements of the feeding vessel, and 65% more vascular arches among true positive glands than the high frequency probe Fig. 3.

**Table 2:** Number of visible vascular features for ultrasound true and false positive glands according to probe

Conventional probe			OR [95% CI]	Vascular probe			OR [95% CI]	Conventional vs. vascular probe: Visibility of vascular features of ultrasound true positive glands			Wilcoxon signed rank test
Visible vascular features	TP	FP	2.53 [1.62–3.97]	Visible vascular features	TP	FP	1.94 [1.26–3.00]	Visible vascular features	Conv. probe	Vascular probe	<0.0001
0	24	20		0	7	7		0	25	8	
1	22	2		1	21	4		1	22	21	
2	43	7		2	39	11		2	44	39	
3	27	1		3	49	4		3	27	50	
SUM	116	30		SUM	116	26		SUM	118^a^	118^b^	

^a,b^Including 2 lesions which were evaluated for vascularity by both probes, but which were only correctly identified as positive by the opposite probe

*OR* odds ratio, *Conv.* conventional

**Table 3:** Visibility of individual vascular features of ultrasound true positive glands according to ultrasound probe

Feeding vessel	Conventional probe Visible	Conventional probe Not visible	Exact McNemar
Vascular probe Visible	89	19	<0.0001
Vascular probe Not visible	1	9	

Polar placement of feeding vessel	Conventional probe Visible	Conventional probe Not visible	Exact McNemar
Vascular probe Visible	64	21	0.0003
Vascular probe Not visible	3	30	

Vascular arch	Conventional probe Visible	Conventional probe Not visible	Exact McNemar
Vascular probe Visible	30	26	0.0001
Vascular probe Not visible	4	58	

*n* = 118 glands evaluated for vascularity, including 2 lesions per probe which were evaluated for vascularity by both probes, but which were only correctly identified as positive by the other probe

### Effect of vascular features on confidence score

For both probes, a Kruskal-Wallis test indicated that at least one level of vascular features had an effect on the confidence score (*p* = 0.0001). A post hoc Cuzick test showed a statistically significant trend for increasing confidence scores across the four levels of vascular features (*p* < 0.0001, Table 4). However, the diagnostic confidence score for true positive glands did not differ significantly between the probes (Wilcoxon signed rank test *p* = 0.11).

**Table 4:** Confidence score versus number of vascular features for ultrasound true positive glands

CONVENTIONAL PROBE	Number of vascular features					Kruskal-Wallis test	Cuzick’s test for trend
**Confidence score**	**0**	**1**	**2**	**3**	**Total**	0.0001	<0.0001
**1 Low**	**6**	**6**	**5**	**1**	**18**		
**2 Medium**	**15**	**8**	**19**	**4**	**46**		
**3 High**	**3**	**8**	**19**	**22**	**52**		
**Total**	**25**	**22**	**44**	**27**	**116**		

VASCULAR PROBE	Number of vascular features					Kruskal-Wallis test	Cuzick’s test for trend
**Confidence score**	**0**	**1**	**2**	**3**	**Total**	0.0001	<0.0001
**1 Low**	**3**	**4**	**5**	**2**	**14**		
**2 Medium**	**4**	**12**	**18**	**11**	**45**		
**3 High**	**0**	**5**	**16**	**36**	**57**		
**Total**	**8**	**21**	**39**	**50**	**116**		

## Discussion

Previous studies have shown that ultrasound is a valid first line test for localizing enlarged parathyroid glands in patients with primary hyperparathyroidism [13, 21]. In this context, optimizing the conventional ultrasound examination is a logical next step. To our knowledge, this is the first study comparing the visibility of vascular features in hyperfunctioning parathyroid glands using different ultrasound probes.

The most common mimics of hyperfunctioning parathyroid glands on ultrasound are cervical lymph nodes and thyroid nodules [22, 23]. These structures, however, may be distinguished from hyperfunctioning parathyroid glands by their vascular patterns [22]. Lymph nodes have a central vascular hilum, whereas thyroid nodules show peri- and intralesional vascularization from an internal thyroid vessel [22, 24]. Hyperfunctioning parathyroid glands, on the other hand, typically display a prominent extrathyroidal polar feeding vessel and a peripheral vascular arch [10].

In this study, we showed that the addition of a low-frequency vascular transducer significantly increased the number of detectable vascular features in pathological parathyroid glands compared to conventional ultrasound employing a high-frequency probe. Additionally, the odds of a gland being true positive increased with the number of visible vascular features. This finding is in line with a study by Lane et al., which found that the presence of an extrathyroidal feeding vessel increased the sensitivity for detecting parathyroid adenomas from 73% to 83% [25]. Similarly, a prospective study from 2003 reported a statistically significant increase in diagnostic accuracy when a feeding vessel was identified [26]. Even when it comes to the challenging detection of intrathyroidal parathyroid adenomas, a retrospective study from 2013 found a strong correlation between the presence of a polar feeding vessel and correct identification of an intrathyroidal parathyroid adenoma on ultrasound [27]. Consequently, the addition of a vascular probe to the conventional ultrasound examination could potentially help reduce the number of false positive findings on ultrasound and/or increase the sensitivity for detecting pathological parathyroid glands [26, 28].

While our exploratory analysis showed a statistically significant trend for increasing diagnostic confidence scores concurrently with increasing numbers of visible vascular features, there was no statistically significant difference between the confidence scores obtained with each probe. The reason for this is likely three-fold. First, the sample size was not designed to compare the confidence scores between the ultrasound probes. Second, by design the examiners had no option of indicating an improved diagnostic confidence with the addition of the vascular probe in cases where they had already given a maximum confidence score based on the conventional probe alone. Third, the examiners in this study were all experienced in parathyroid ultrasound. It may well be that less experienced examiners would benefit even more from the increased visibility of parathyroid vascular features [29].

Some limitations should be addressed. The idea for the present study arose during the pilot phase of a larger cohort study. The sample size and design were therefore constrained by a predefined protocol. This restricted the recording of diagnostic confidence, as mentioned above, but it did not influence the notation of the number of vascular features. Because the number of vascular features is an objective measure, whereas diagnostic confidence is subjective, we considered the number of visible vascular features to be the most reliable/reproducible measure of the effect of adding a vascular probe. Finally, we cannot be certain of the generalizability of our results to ultrasound probes from other manufacturers.

Future studies could aim to investigate the impact of the addition of a low frequency vascular probe on both the sensitivity of the ultrasound examination, the diagnostic confidence of the examiner, and the interobserver variability. Furthermore, the generalizability of our results to probes from other manufacturers should be investigated. In centers that employ upfront ultrasound, it would be interesting to examine the effect of a second-look ultrasound examination on the diagnostic sensitivity in cases where the initial ultrasound examination is negative, or the ultrasound examiner’s confidence in the result is low. Such an approach could potentially spare an even larger number of patients from radiation-based parathyroid imaging while making better use of the finite resources of the healthcare system [29, 30].

In conclusion, we have shown that the addition of a low frequency vascular probe increases the number of visible vascular features in hyperfunctioning parathyroid glands, which aids in their preoperative detection. Whether or not this can increase the diagnostic confidence of ultrasound examiners has yet to be substantiated.

## Supplementary Information

Supplementary material
