# Supplementary material for: The detection of pathological parathyroid glands is facilitated by identifying vascular features on ultrasound: the potential benefit of a low-frequency vascular probe
Source: Endocrine. 2024 Aug 8;86(3):1131–9. doi: 10.1007/s12020-024-03986-y (PMC11554940; doi:10.1007/s12020-024-03986-y)
Supplement: Supplementary file 1 — Supplementary material [file 12020_2024_3986_MOESM1_ESM.docx]

**Supplementary material**

| Online Resource 1: Measures of diagnostic accuracy | | | | |
| --- | --- | --- | --- | --- |
| **LOCALIZING SCANS ONLY** | | | | |
| **Characteristic** | **Conventional probe** | | **Conventional + vascular probe** | |
| No. of patients | 124 | | 121 | |
|  | Value | 95% CI | Value | 95% CI |
| Sensitivity per patient (≥1 true positive quadrant per patient) | 89.5% | 82.7-94.3 | 91.7% | 85.3-96.0 |
| Sensitivity per quadrant | 81.7% | 73.9-89.4 | 83.5% | 75.7-91.2 |
| Specificity per quadrant | 91.5% | 88.5-94.5 | 92.5% | 89.5-95.4 |
| PPV per quadrant | 79.5% | 73.0-85.9 | 81.7% | 75.3-88.1 |
| NPV per quadrant | 92.6% | 89.0-96.2 | 93.3% | 89.7-96.9 |
| **ALL SCANS (localizing and nonlocalizing)** | | | | |
| **Characteristic** | **Conventional probe** | | **Conventional + vascular probe** | |
| No. of patients | 136 | | 136 | |
|  | Value | 95% CI | Value | 95% CI |
| Sensitivity per patient (≥1 true positive quadrant per patient) | 81.6% | 74.1-87.7 | 81.6% | 74.1-87.7 |
| Sensitivity per quadrant | 73.4% | 65.1-81.8 | 73.4% | 65.1-81.8 |
| Specificity per quadrant | 92.2% | 89.5-95.0 | 93.3% | 90.6-95.9 |
| PPV per quadrant | 79.5% | 73.0-85.9 | 81.7% | 75.3-88.1 |
| NPV per quadrant | 89.4% | 85.6-93.3 | 89.6% | 85.7-93.4 |
